# Dual mobility total hip replacement in a high risk population

**DOI:** 10.1051/sicotj/2016037

**Published:** 2016-12-07

**Authors:** Jatinder Singh Luthra, Amur Al Riyami, Mohamad Kasim Allami

**Affiliations:** 1 Khoula Hospital PO Box 90 PC 116 Mina Al Fahal Oman

**Keywords:** Dual mobility, Total hip replacement, Dislocation

## Abstract

*Objective*: The purpose of the study was to evaluate results of dual mobility total replacement in a high risk population who take hip into hyperflexed position while sitting and praying on the floor.

*Method*: The study included 65 (35 primary total replacement and 30 complex total hip replacement) cases of total hip replacement using avantage privilege dual mobility cup system from biomet. A cemented acetabular component and on femoral side a bimetric stem, either cemented or uncemented used depending on the canal type. Ten cases were examined fluoroscopically in follow up.

*Result*: There was dislocation in one patient undergoing complex hip replacement. Fluoroscopy study showed no impingement between the neck of prosthesis and acetabular shell at extremes of all movements.

*Conclusion*: The prevalence of dislocation is low in our high risk population and we consider it preferred concept for patients undergoing complex total hip replacement.

## Introduction

Instability is an extremely significant cause of morbidity following total hip replacement (THR). The incidence of instability after primary and revision replacement has been reported to be as high as 7% and 25%, respectively [1]. The cumulative risk of first time dislocation is 2% at one year and 7% after 15 years of primary hip replacement [2]. The concept of dual mobility articulation was developed in 1970 by Bousquet to increase the range of motion and to decrease dislocation risk. It combined a small head to decrease wear (low friction arthroplasty principles stated by Charnley [3]) and a large head to increase stability (MacKee and Farrar [4]). Several studies have looked at the outcome of dual mobility articulation in primary THR [5–18] and in revision THR [5, 19–24].

We present our series of cases performed in high risk population whose cultural demand requires sitting on the floor [25].

## Material and methods

This is a retrospective study of 65 patients undergoing complex primary or revision hip replacement for different etiologies in selective cases. The cases included in our study were consecutive cases of dual mobility cup (DMC) done at our institute from June 2010 till July 2014.

**Figure 1.**
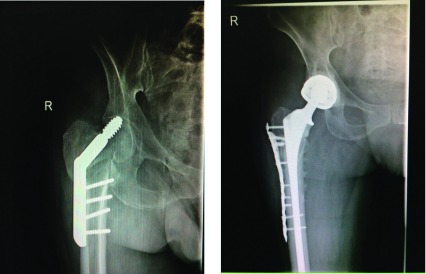
Radiograph of patient with failed DHS revised to dual mobility THR. Locking plate used to stabilize the trochanteric fracture.

**Figure 2.**
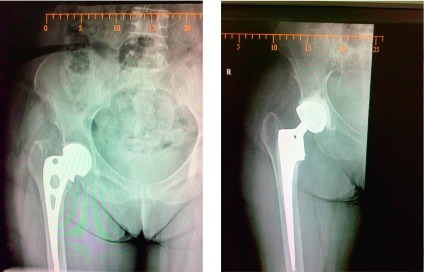
Failed Austin Moore prosthesis managed with calcar bearing dual mobility THR.

The inclusion criteria for a patient undergoing total hip replacement (THR) to have DMC were those at high risk of dislocation. These included patients who were either more than 60 years, had poor soft tissue envelope around the hip, were non compliant, were elderly and had sustained femoral neck fracture, underwent failed hip surgeries, had a septic hip, and had undergone a revision THR irrespective of the cause (Figures 1 and 2).

Surgery was performed in lateral decubitus position using Moore’s (Southern) approach to hip. The acetabular cup was from Biomet (Avantage privilege DMC) and a bimetric femoral stem was used. A calcar bearing stem was used in three cases and a long stem was used in 10 cases to bypass the defect, or stress riser. Three patients with a fracture of the trochanter were stabilized with a distal tibial locking plate. Structural allograft was used for the support of proximal femur in one patient. Palacos cement was used to fix the acetabular cup in 63 cases and 2 cases were uncemented.

Out of 65 cases, 35 were male and 30 female. The age of the patient ranged from 23 years to 91 years with a mean age of 61 years. It was primary hip replacement in 30 cases and revision surgery (complex THR) in 35 cases. The etiologies of primary and revision cases are shown in Tables 1 and 2, respectively. The cases of osteoarthritis, femoral neck fracture, and fracture acetabulum were those of the elderly. The sickler was the youngest patient, who had undergone attempted arthrodiastasis and his soft tissue envelope around the hip was compromised. Thirteen cases had undergone two or more previous surgeries. In one patient, the hip was operated seven times.

**Table 1.** Primary THR 30 cases.

Osteoarthritis	17
Femoral neck fracture	9
Fracture acetabulum	3
Sickle cell disease	1

**Table 2.** Revision surgery 35 cases.

Failed DHS	11
Failed hemiarthroplasty	9
Infection	4
Periprosthetic infection	2
Revision THR	4
Failed osteosynthesis	5

We used Clexane 4000 IU for postoperative deep vein thrombosis (DVT) prophylaxis routinely till the patients were mobilized. Aspirin was not considered a contraindication for surgery, but clopidogrel had to be stopped seven days before surgery. Patients on warfarin were taken up for surgery only after international normalized ratio (INR) was less than 1.5. The sickler patient underwent preoperative exchange transfusion to reduce his hemoglobin S (HbS) to <40. Drain was used routinely for 24–48 h. The patients were mobilized postoperatively on day one after surgery with the help of a zimmer frame with weight bearing as tolerated.

**Figure 3.**
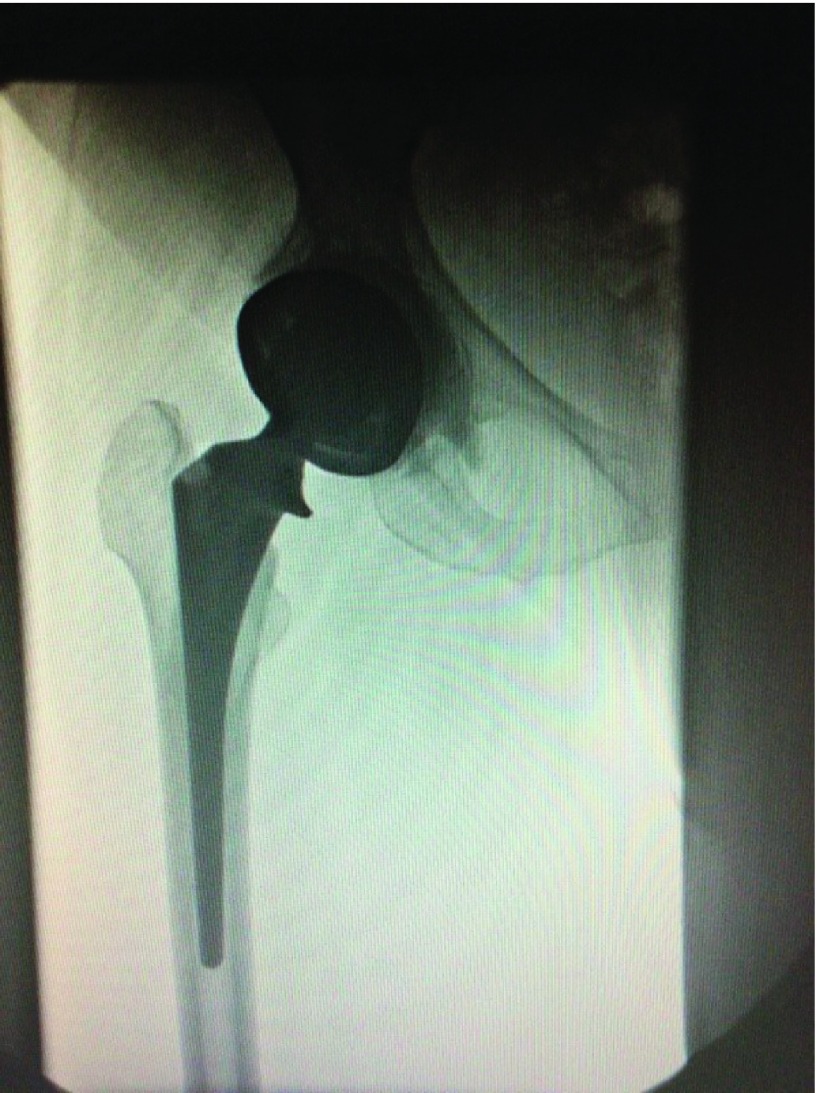
Fluoroscopic examination of a dual mobility THR.

The sutures were removed at two weeks after surgery. X-rays were taken immediately postoperatively, one, three months, and one year after surgery. Ten patients agreed for a fluoroscopic evaluation after surgery (Figure 3).

## Results

The follow-up of our cases has ranged from 18 months to six years with a mean follow-up of five years. There was a minor surgical wound related complication in six patients (three leaky wounds and three bruising under the skin), which settled after the stoppage of Clexane.

There was no dislocation seen in the primary total THR group. In one case of revision hip that was immunocompromised, tramadol addict with hepatocellular carcinoma, there was deep infection and dislocation. His infection could not be controlled despite antibiotics and debridement and he ended up with Girdlestone excision arthroplasty and eventually died due to his medical condition.

There was one mortality in the early postoperative period in an elderly patient who underwent an uncemented THR due to suspected myocardial infarction in the early postoperative period.

X-rays taken during follow-up have not shown any evidence of loosening around the acetabulum.

**Figure 4.**
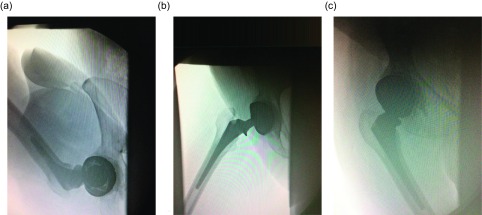
Fluoroscopic examination of a dual mobility THR in (a) flexion, (b) abduction and (c) adduction.

The fluoroscopic evaluation was done for 10 cases. In these cases, a mean flexion of 120°, abduction 30°, and adduction 10° were observed (Figure 4). There was no impingement between the femoral neck and the metallic shell at extremes of movement. The third articulation between the polyethylene (PE) and the neck of femoral prosthesis could not be seen as the liner is radiolucent.

Patients in our series would sit on the floor and pray as a custom in the local population, despite being advised not to do such activities.

## Discussion

In our series, the DMC has been used for selective cases of hip replacement, which were at high risk of postoperative instability. The indication of DMC included patients who were either more than 60 years, were non compliant with a history of substance abuse, who had a history of prior hip surgery, had a compromised soft tissue envelope around the hip, and who were elderly and had sustained a femoral neck fracture. Our early results with these implants have shown a 98% survivorship at a mean follow-up of five years without implant loosening. We have had no dislocations in our primary THR with DMC. Studies have shown a dislocation rate of 0–3.6% in primary THR [6, 7, 9, 26].

At 10 years the survivorship has ranged from 93 to 99% [10, 17], with one long-term study showing 80% survivorship at 22 years [7].

In the case of complex THR, the dislocation rate ranged from 5% to 30% because of the bone loss, compromised muscles, and soft tissues around the hips. The use of DMC in complex THR has shown the dislocation rate to range from 1% to 10% at eight year follow-up [5, 18, 20, 21, 24, 27]. The implant survivorship has shown to be in the range of 95.6–96.2% at 3–8 years [5, 18, 20, 27]. We have used the DMC in 30 cases of complex THR and have had dislocation in one case.

The case of a septic hip undergoing THR had compromised soft tissues and has increased the chances of postoperative instability [28]. We have used such cups in four septic cases undergoing staged revision. The dislocation reported in our series has been shown in one such case. The patient had a history of substance abuse and was immunocompromised with hepatocellular carcinoma and suffered from postoperative deep infection. He dislocated his hip and the periprosthetic infection could not be controlled with antibiotics and debridements and he ended up with Girdlestone excision arthroplasty.

Elderly patients with a femoral neck fracture have improved hip scores and better functional results after the THR [29]. There are higher postoperative dislocation rates following the THR after femoral neck fracture, which is almost five times higher than that reported for THR after osteoarthritis, meta-analysis has shown dislocation rates of 10.7% [30]. A randomized control trial comparing the internal fixation with THR in 100 patients found a dislocation rate of 22% in patients undergoing THR [29]. The use of DMC for THR in the case of a femoral neck fracture has shown a dislocation rate of 1.4% [31]. A comparison of dislocation rates has been done for conventional hip replacement and DMC replacement; there was a postoperative dislocation incidence of 14.3% in a conventional total hip and no dislocation was observed in the dual mobility group [32]. There were nine patients with femoral neck fracture, in our series five patients had failed osteosynthesis and four underwent THR primarily and no postoperative dislocation occurred. These patients sit on the floor and continue to pray on the floor as demanded by local customs.

Intraprosthetic dislocation (IPD) is peculiar to the DMC [33]. It occurs between the smaller head and polyliner due to a “bottle opener” effect. One has to be aware of the condition in order not to miss it which results in excessive metallosis and failure of the DMC [34]. The proposed theory causing IPD is the wear of PE retentive chamfer [7]. The head lies asymmetrically in the cup and might be mistaken for polywear. The dislocated liner has been described as a bubble sign and is pathognomic of intraprosthetic dislocation. The incidence of IPD in newer designs is probably lower because of polished neck and reduced wear of the liner at third articulation. In our mean follow-up of five years, we have not encountered this complication.

To the best of our knowledge, there has been no study in the literature evaluating these cups fluoroscopically. We have studied 10 cases fluoroscopically. There was no impingement between neck and metal cup at extremes of movement. It was difficult to comment on the articulation between the neck of the femoral prosthesis and the polyliner (third articulation) as it could not be seen due to the liner being radiolucent.

The addition of a radiopaque marker in the polyliner can help to evaluate movement at this articulation. The presence of this marker can also help in diagnosing IPD.

In younger patients, these cups should be used with caution as they are high demand cases and have high chances of PE wear and higher incidence of IPD [18, 27]. In our series, there were five patients under 40 years and the youngest was a sickler 23 years old who had arthrodiastasis done and had compromised soft tissue.

Ninety-six percent (96%) of the dual mobility cups were cemented and no early radiographic loosening was seen at a mean follow-up of five years.

The limitation of our study that it is a retrospective study with a maximum follow-up of six years. The pre- and postoperative scoring with a validated hip scoring system which takes into account the lifestyle of our cohort of population, whose activities of daily living involve sitting on the floor not wearing shoes, would have helped in providing more information with functional results with these implant systems.

## Conclusion

The DMC is an effective solution for the management of high risk cases undergoing total hip replacement for different causes to reduce the incidence of postoperative instability. The modification of a liner may be helpful to evaluate the system to diagnose the IPD. An alternate scoring system that takes into account different cultural aspects will help in the objective evaluation and comparison of results in different subsets of the population. A larger study group with a longer follow-up in randomized trials and meta-analysis are required to further confirm these findings.

## Conflict of interest

The authors declare no conflict of interest in relation with this paper.
